# “Does My Teacher Believe I Can Improve?”: The Role of Meta-Lay Theories in ESL Learners’ Mindsets and Need Satisfaction

**DOI:** 10.3389/fpsyg.2020.01417

**Published:** 2020-08-07

**Authors:** Nigel Mantou Lou, Kimberly Ann Noels

**Affiliations:** Department of Psychology, University of Alberta, Edmonton, AB, Canada

**Keywords:** meta-lay theory, self-determination, language mindsets, feedback, language learning, English as a second language

## Abstract

Supporting students’ growth mindsets (i.e., beliefs that ability can be improved) and basic psychological needs (i.e., needs for autonomy, competence, and relatedness) is an important way to sustain their motivation and resilience after challenging situations. We argue that others’ feedback may support or undermine mindsets and need satisfaction simultaneously through students’ meta-lay theories—that is, students’ perceptions of whether others (in this case, their teacher) believe that ability can be improved or not. We conducted a randomized controlled experiment in which 180 university students who spoke English as their second language failed a difficult English test and received either feedback from a teacher who consoled their lack of ability, feedback that focused on improving ability, or no feedback. We found that compared to students receiving no feedback, students receiving ability-consoling feedback perceived that the teacher believed less in their potential and felt less competent, and students receiving improvement-oriented feedback perceived that the teacher believed more in their potential. Consequently, meta-lay theory (“the teacher believes I can change my ability”) predicted students’ endorsement of growth mindsets (“I believe I can improve”) and need satisfaction (sense of competence, relatedness, and autonomy). In turn, mindsets and need satisfaction jointly predicted language confidence and beliefs about mistakes. Only need satisfaction, however, predicted task avoidance and duration of task engagement. Meta-lay theories underlie the processes through which feedback supports or undermines students’ resilience after failure.

## Introduction

When learning and using a new language, learners often experience difficulties and setbacks, such as making mistakes in writing, miscommunicating with others, getting bad grades, and being ignored by interlocutors because of accents/lack of proficiency. These experiences can undermine learners’ confidence to use the language and motivation to continue second language learning. Prior research indicates that supporting learners’ growth mindsets (i.e., beliefs that ability can be improved; Dweck et al., 1995) and basic psychological needs (i.e., needs for autonomy, competence, and relatedness; Ryan and Deci, 2020) is an important way to help them sustain their motivation and resilience. On the one hand, learners with growth (vs. fixed) mindsets tend to adopt mastery goals that focus on improvement, to seek challenges, and to react positively to language failures (Lou and Noels, 2016, 2020; Dweck and Yeager, 2019). On the other hand, learners who are satisfied with their psychological needs feel more intrinsically motivated, enjoy challenges more, and persist longer in language learning (Noels, 2001; Oga-Baldwin et al., 2017; Noels et al., 2019). Strategies for supporting learners’ growth mindsets (Park et al., 2016) and need satisfaction (Jang et al., 2016) are important resources for instructional design and teaching practice that support learners’ engaged and successful learning (see also Linnenbrink-Garcia et al., 2016).

Despite the importance of fostering growth mindsets and need satisfaction, little attention has been directed toward understanding how these two frameworks are related and whether the effects they each predict are shaped through a similar social–psychological mechanism. Mindset theory (MT) and research emphasize how learners develop fixed mindsets (i.e., beliefs that intelligence is immutable) and growth mindsets (i.e., beliefs that intelligence can be cultivated), as well as the consequences on motivation and achievement of holding different mindsets (Haimovitz and Dweck, 2017). On the other hand, self-determination theory (SDT) focuses on how learning conditions meet learners’ basic psychological needs as well as how the need satisfaction predicts self-determined motivation, achievement, and well-being (Ryan and Deci, 2020). The connections between the two frameworks are important because each of these theories offers insights into motivation and self-regulation and because empirical research shows each of these theories predicts achievement and important psychological outcomes (Burnette et al., 2013; Ryan and Deci, 2020). One goal of this study is to integrate these frameworks by examining what types of feedback influence learners’ growth mindsets and need satisfaction. We identify a key psychological factor—meta-lay theory (i.e., perceptions of whether others believe one’s ability can be improved or not; Rattan et al., 2018), and we argue that the meta-lay theory shares a common mechanism through which teachers’ supportive feedback influences learners’ mindsets and need satisfaction. Another goal of this study is to examine whether growth mindsets and need satisfaction make distinct contributions to learners’ responses to failure. Answering these questions can bridge the connections between MT and SDT, extending the understanding of motivational processes by integrating both theories. Such amalgamation can also offer insights for educational practice that supports learners’ resilience and success.

### Ability Feedback in Self-Determination Theory and Mindset Research: The Role of the Meta-Lay Theory

Teachers’, parents’, and peers’ feedback can support learners’ motivation and influence their reaction to failure situations, which in turn shapes learners’ subsequent resilience (Schunk, 1983; Rattan et al., 2012; Ahn et al., 2016; Fong et al., 2019; Zhou et al., 2019). For example, feedback that encourages learners to improve their skills can buffer the negative effect of failures on perceived competence (see Fong et al., 2019, for a meta-analysis). The importance of growth-oriented feedback is emphasized in both SDT and MT. SDT conceives humans as growth-oriented organisms and emphasizes that nurturing conditions should meet people’s fundamental needs for competence, relatedness, and autonomy (Deci and Ryan, 2000; Skinner et al., 2008; Jang et al., 2016; Vansteenkiste et al., 2018). If learners receive feedback and opportunities to experience growth, the learners feel more satisfied with their psychological needs and more self-determined to initiate learning (Noels, 2001; Skinner et al., 2008; Ruzek et al., 2016; Oga-Baldwin et al., 2017; Lou et al., 2018; Burns et al., 2019). On the other hand, MT emphasizes that beliefs about growth shape human development and that learning contexts and socialization can modify these basic beliefs about their ability (Rattan et al., 2012; Haimovitz and Dweck, 2017; Barger, 2019). Learners who receive encouragement for improvement (e.g., praise for effort rather than ability and encourage learners to make mistakes) are more likely to endorse growth mindsets about their ability, to see challenges and making mistakes as learning opportunities, and to put effort into overcoming challenges (Leith et al., 2014; Haimovitz and Dweck, 2017).

Both MT and SDT emphasize that feedback is an important interpersonal process that impacts motivation, but it is unclear whether there are shared social processes that influence both mindsets and need satisfaction. We propose that learners’ meta-lay theories underlie the social–psychological processes through which learners perceive and react to others’ feedback. Rattan et al. (2018) argued that “just as people have their own lay theories or mindsets, they may be aware that others hold such beliefs as well” (p. 55). Of particular relevance to learning are the beliefs that significant others hold about the learner. In this research, we use the term “meta-lay theories” to refer to learners’ perceptions about whether the feedback providers believe the learners’ ability is fixed (i.e., fixed meta-lay theories) and malleable (i.e., growth meta-lay theories). Specifically, through interactions with others (e.g., receiving feedback), learners may see themselves and develop their mindsets (Haimovitz and Dweck, 2017). This perspective is in line with a long tradition of research that demonstrated that learners’ beliefs are influenced by teachers’ expectations and feedback (Friedrich et al., 2015; Rubie-Davies et al., 2015). Learners not only are aware of others’ expectations but also often assess their own ability and potential by “reflecting” how others think of them (Wigfield and Eccles, 2000; Bouchey and Harter, 2005). As such, learners may internalize others’ expectations/beliefs (e.g., “Does my teacher think that I am good at math?”) to their own self-concepts of ability (e.g., “Am I good at math?”; Wigfield and Eccles, 2000; Lazarides and Watt, 2015). Similarly, we argue that learners can also perceive others’ lay beliefs (meta-lay theories; e.g., “Does my teacher think that I can improve my ability?”) and internalize others’ beliefs to their own mindsets about ability (e.g., “Can I improve my ability?”; Dweck et al., 1995).

#### Ability-Consoling and Improvement-Oriented Feedback to Failures

Students are motivated to understand what others think about them and their ability, and one source of information is others’ responses to the learners’ learning outcomes (Swann, 1983; Bouchey and Harter, 2005). In this study, we focus on ability-consoling and improvement-oriented feedback because they are commonly used to comfort and to encourage struggling learners (Rattan et al., 2012; Burns et al., 2019). After failure, some people may comfort learners by assuring them that they are competent in other domains. They might say, for example, “Some people aren’t naturally good at languages. But they are good at other things, such as math.” In contrast, some people might focus their feedback on encouraging the learners to improve their ability, saying, “If you keep working on it, you’ll improve your ability.” These conceptualizations of the ability-consoling and improvement-oriented feedback are in line with a previous study (Rattan et al., 2012; Study 4). Rattan et al. (2012) showed that learners who received ability-consoling feedback perceived their teachers to have a stronger fixed mindset than those who received improvement-oriented feedback. We further argue that this feedback may not only signal that the feedback providers have a fixed or growth mindset themselves, but also convey messages about whether the feedback providers believe in the learners’ potential to improve or not, thus affecting learners’ own mindsets. Moreover, this feedback may foster or thwart learners’ need satisfaction.

Ability-consoling feedback may at first seem to restore learners’ general sense of competence by assuring learners’ competence in other domains, but it poses a static, immutable view of ability and signals that the feedback providers do not have a high expectation that the learners can change in the target domain (see Rattan et al., 2012). Learners may perceive that the feedback provider does not believe that their ability can be improved (fixed meta-lay theories). From an SDT perspective, ability-consoling feedback can be construed to be controlling instructional behaviors that signal that the learners have little control or competence over their learning, which may also undermine learners’ sense of autonomy (i.e., perceived volition and psychological freedom; Reeve and Jang, 2006; Jang et al., 2016). Ability-consoling feedback may also influence how learners feel about their relatedness to the feedback providers (Niemiec and Ryan, 2009). Research demonstrated that after failure, learners viewed the teacher–student relationship more negatively (i.e., decreased in sense of relatedness) when teachers provided feedback that indicated learners had a fixed ability than when teachers provided no feedback (Skipper and Douglas, 2015).

In contrast, improvement-oriented feedback that focuses on the learners’ growth may indicate that the feedback provider believes that the learners have the potential to grow and can become effective in the task (Rattan et al., 2012; Burns et al., 2019). Thus, improvement-oriented feedback supports both the endorsement of growth mindsets (Dweck et al., 1995) and a sense of competence (Deci and Ryan, 2000; Niemiec and Ryan, 2009).

### Joint Effects of Mindsets and Need Satisfaction

Although both SDT and MT each provide insight into learners’ motivation and achievements, little empirical research has addressed their connections and unique contributions simultaneously. One reason for this lack of synthesis is that they come at motivation from different traditions. Specifically, need satisfaction influences learners’ effort and engagement through internalizing and integrating the values of learning activities into personal relevance/meaning (Noels et al., 2016; Vansteenkiste et al., 2018). For example, learners whose needs are satisfied are more likely to engage in goal pursuits for self-determined reasons and thus invest more and persist in goal pursuits (Oga-Baldwin et al., 2017; Ryan and Deci, 2020). In contrast, mindsets are argued to influence effort and self-regulation, providing a lens through which people can make sense of their learning environment and ability (Molden and Dweck, 2006; Lou et al., 2017). For example, learners with fixed (vs. growth) mindsets are less likely to persist and more likely to feel ego-threatened after setbacks because they tend to attribute failures and mistakes to a lack of ability (vs. lack of effort). In summary, learners are driven by their need satisfaction but are also constantly trying to construct meaning out of their learning experiences, and therefore both processes are important for predicting learners’ motivation and behaviors (Dweck, 2017). SDT and MT encompass different components of motivational processes that can operate simultaneously and complement each other in predicting adaptive motivational tendencies.

### The Current Study

**FIGURE 1:**
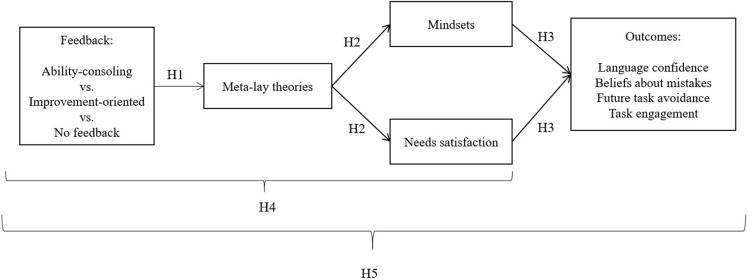
Hypothesized path model.

In this study, we integrate SDT and MT through understanding not only their shared contextual and cognitive antecedents (i.e., how ability-consoling vs. improvement-oriented feedback and meta-lay theories influence both growth mindsets and need satisfaction) but also the connection between these constructs and their joint predictions on learners’ self-regulation, adaptive beliefs, and emotional responses to failure situations. We proposed a theoretically based mediational model (Figure 1). Specifically, we argue that cues in the learning environment, such as messages that are focused on existing talent or future improvement, affect learners’ meta-lay theories, which in turn predict one’s need satisfaction and mindset. As a result, learners’ mindsets and need satisfaction jointly predict motivational outcomes.

This study focused on the context of migrant learners’ English learning and their motivation after receiving different feedback. Migrant learners often receive comments and feedback about their language ability in everyday communication (Lippi-Green, 1997). As a result, some English-as-a-second-language (ESL) learners believe that making mistakes in English can make them look “dumb” (i.e., negative view of mistakes; Tulis et al., 2018); spend less time learning from mistakes; avoid situations where they fail (e.g., task avoidance goals, Elliot and Church, 1997; Lou and Noels, 2019a); and lack confidence when using English (Clément and Kruidenier, 1985; Noels et al., 1996; Lou and Noels, 2018). How ESL learners respond to language failures can profoundly affect their social adaptions and academic growth. Thus, one goal of this study is to understand how learners’ perceptions about feedback providers can support or hinder learners’ language confidence and anxiety (i.e., affective outcome), beliefs about mistakes (i.e., cognitive/belief outcome), task avoidance goal (i.e., behavioral tendency outcome), and time spent on learning from mistakes (i.e., objective behavioral outcome) after failing an English test. Based on our theoretical mediational model, we proposed five hypotheses:

H1: Receiving ability-consoling feedback predicts fixed meta-lay theories (e.g., “I think the teacher believes that I cannot improve my language ability”), whereas receiving improvement-oriented feedback predicts growth meta-lay theories (e.g., “I think the teacher believes that my language aptitude is changeable”).

H2: Fixed (vs. growth) meta-lay theories predict more subjective support for psychological needs (autonomy, competence, and relatedness) and stronger endorsement of a growth (vs. fixed) mindset about language learning.

H3: Fixed (vs. growth) mindsets and need satisfaction jointly predict adaptive outcomes. Fixed mindsets would negatively predict ESL learners’ language confidence, beliefs about mistakes, task avoidance, and duration of task engagement, whereas need satisfaction would positively predict these outcomes.

H4: The effects of ability-consoling and improvement-oriented feedback on language mindsets and need satisfaction are mediated by meta-lay theories.

H5: Feedback influences motivational outcomes through meta-lay theories and then mindsets and need satisfaction.

## Materials and Methods

### Participants

Participants were recruited through a psychology research pool in a large Canadian university where English is the dominant language. Ethical permission for this study is approved by the University of Alberta’s human research ethics office. Students who self-identified as foreign-born and spoke English as a second language could sign up for this study in exchange for course credits. All participants passed the university’s English requirement or an equivalent in an ESL test [90 in Test of English as a Foreign Language (TOEFL) or 6.5 in International English Language Testing System (IELTS)]. We recruited 192 eligible participants. Five participants who did not fill out the key measures due to procedural errors were not included. Seven participants were also excluded either because they knew the purpose of the study was about the impact of the teacher/feedback or because they did not pay attention to the feedback (indicated during debriefing). The final sample was comprised of 180 participants (60.6% female)^1^. Their ages ranged from 15 to 26 years old (*M* = 19.68; *SD* = 2.60). Participants are either international students (*n* = 74) or immigrants (*n* = 106), and they had lived in Canada for 5.89 years on average (*SD* = 4.69). Most of them originated from Asian countries (80.0%; see Supplementary Data Sheet 1 for details).

### Procedure and Manipulation

Participants waited outside of a research lab in groups of two to four. The experiment was conducted by one of the two experimenters (one male and one female) who were blind to the research question and greeted the participants. The experimenters were Anglo-Canadian and were dressed professionally to convey the appearance of an English teacher. Before proceeding with the explanation of the task, the experimenter introduced himself/herself as an English teacher who was working on his/her master’s degree in education (see Supplementary Data Sheet 1 for the script the experimenter used). Participants signed a consent form that included a statement of the purpose of the research. Participants were told that the study examined psychological factors related to learners’ language ability and performance on an English test. The experimenter also explicitly told the participants that they could quit anytime during the experiment.

Next, the experimenter instructed the participants to complete an English test with a time limit of 15 min. The English test was comprised of (a) five fill-in-two-to-three-blank questions from a graduate record examination (GRE) practice exam of English verbal ability and (b) one passage of text, followed by eight reading comprehension questions from a Law School Admission Test (LSAT) practice exam (see Supplementary Data Sheet 1 for English test items). A few minutes after completing the test, the participants were then informed by the “teacher” through the computer that they failed the test, accompanied by one of the three feedback conditions: no feedback (i.e., control condition), ability-consoling feedback, or improvement-oriented feedback (with the differences bolded). Ability-consoling and improvement-oriented feedback scripts were adapted from a previous study (Rattan et al., 2012), which showed different effects on learners’ perceptions of their teachers’ mindsets.

Ability-consoling feedback: “I’m sorry that you did not do well on the test. I wanted to let you know that you’re an adept and capable student. *English isn’t a subject for everyone—it’s okay if you didn’t do as great as you hoped. Some people aren’t naturally good at languages. But I’m sure you have great talent in other subjects.* I care about how you’re doing and feeling with this task, so if you have any questions, feel free to talk to me about the task or about language learning in general after the study.”

Improvement-oriented feedback: “I’m sorry that you did not do well on the test. I wanted to let you know that you’re an adept and capable student. *Like with many things, practice makes perfect. If you put in the work, you’ll be at the level of proficiency that you want, so keep working on it.* I care about how you’re doing and feeling with this task, so if you have any questions, feel free to talk to me about the task or about language learning in general after the study.”

After reading the feedback, participants filled out a questionnaire containing the measures described below. To encourage participants’ candid responses, the teacher informed the participants that the teacher had no access to the questionnaire answers. At last, participants were offered to review the test questions and to learn from the answer keys. The computer automatically recorded the time participants stay on this learning task. After the experiment, the experimenter asked participants about their thoughts on the study’s purpose and the feedback. Finally, the experimenter offered all participants a debriefing letter, including the purpose of the study, and fully debriefed them verbally.

#### External Manipulation Check

To ensure that the feedback would induce different perceptions about the feedback provider, a separate sample of students from the same university (*n* = *39*) were asked to imagine that they were ESL learners who had taken an English test and received feedback from a teacher. Participants were then randomly assigned to either ability-consoling or improvement-oriented (see Supplementary Data Sheet 1). All participants were given two items to measure their perceptions about the teachers’ consoling intention (Do you agree that the teacher’s intention is to console the student for failing the test?) and improvement intention (Do you agree that the teacher’s intention is to encourage the student to improve?) on a five-point scale (1 = Strongly Disagree to 5 = Strongly Agree). We ran a 2 × 2 mixed-model ANOVA. We found a significant interaction effect, *F*(1,37) = 82.28, *p* < 0.001, η_*p*_^2^ = 0.69 (strong effect). Specifically, participants in the consoling condition rated the teacher’s consoling intention (M = 4.19, SD = 0.60) more strongly than his/her improvement intention (M = 2.38, SD = 1.20), *F*(1,20) = 39.03, *p* < 0.001, η^2^ = 0.66 (strong effect). Those in the improvement condition rated the teacher’s improvement intention (M = 4.61, SD = 0.61) more strongly than the consoling intention (M = 3.11, SD = 1.02), *F*(1,17) = 55.08, *p* < 0.001, η_*p*_^2^ = 0.76 (strong effect). Furthermore, participants in the consoling condition believed that the teacher was more consoling than did participants in the improvement condition, *F*(1,37) = 11.29, *p* < 0.001, η_*p*_^2^ = 0.31 (strong effect), whereas participants in the improvement condition believed that the teacher was more encouraging of improvement than did participants in the consoling condition, *F*(1,37) = 50.63, *p* < 0.001, η_*p*_^2^ = 0.58 (strong effect). These results validated that the feedback message can induce the corresponding perceptions about the feedback provider.

### Questionnaire

The descriptions of each measure, including mean (*M*), standard deviation (*SD*), skewness, kurtosis, and Cronbach’s alpha (α), are reported in Table 1 (see Supplementary Data Sheet 1 for all items). All measures used a five-point scale ranging from 1 (Strongly Disagree) to 5 (Strongly Agree), unless otherwise stated.

**TABLE 1:** Descriptive statistics and correlations among key variables.

	1	2	3	4	5	6	7	8	9	10	11
1. Meta-lay theories (fixed vs. growth)		−0.18*	−0.25***	−0.31***	0.28***	−0.27***	0.08	–0.06	–0.05	–0.11	–0.10
2. Competence		−	0.55***	0.34***	−0.27***	0.30***	−0.56***	0.46***	0.17*	0.19*	0.17*
3. Autonomy			−	0.35***	−0.25***	0.39***	−0.37***	0.34***	0.12	–0.05	0.21**
4. Relatedness				−	−0.30***	0.23**	−0.24***	0.27***	0.25***	0.05	0.14
5. Language mindsets (fixed vs. growth)					−	−0.32***	0.25***	−0.28***	–0.04	0.08	–0.11
6. Beliefs about mistakes						−	–0.13	0.41***	0.22**	0.02	0.07
7. Future task avoidance							−	−0.36**	–0.09	−0.24***	–0.06
8. Language confidence								−	0.16*	0.08	0.31***
9. Duration of task engagement (log transformed)									−	0.04	0.07
10. Gender (0 = women; = men)										−	–0.06
11. Length of residence (year)											−

*M*	2.68	3.29	3.32	3.82	2.76	4.54	2.89	4.50	1.73	0.39	5.89
*SD*	0.88	0.78	0.68	0.64	0.67	0.90	1.04	1.03	0.38	0.49	4.69
α	0.86	0.78	0.70	0.67	0.86	0.89	0.84	0.94	NA	NA	NA
Skewness	0.85	–0.04	0.12	–0.68	–0.02	–0.43	–0.01	–0.51	0.12	0.44	0.71
Kurtosis	1.85	–0.45	–0.29	1.47	–0.20	0.92	–0.87	0.07	0.03	–1.83	–0.41
Theoretical range	1–6	1–5	1–5	1–5	1–6	1–6	1–5	1–6	NA	NA	0–18

***p < 0.001, **p < 0.01, and *p < 0.05.

#### Meta-Lay Theories

The Meta-Lay Theories Scale (Rattan et al., 2018) was adapted to measure participants’ perceptions of whether teachers believe one’s language learning ability can be improved or not. It contained six items with statements such as “The teacher believes that I can always improve my foreign language ability” and “The teacher believes that I can’t really change my language intelligence.” Participants rated their agreement on a six-point scale (1 = Strongly Disagree to 6 = Strongly Agree). An exploratory factor analysis yielded a one-factor solution (extraction based on Eigenvalue > 1; all factor loadings were above 0.62), which explained 58.18% of the variance. We also found that the internal consistency of the scale is high (α = 0.86). A higher score indicated a stronger agreement with fixed (vs. growth) meta-lay theories.

#### Basic Psychological Need Satisfaction

The Basic Psychological Need Satisfaction (BPNS) was used to assess learners’ general satisfaction of their need for autonomy, competence, and relatedness (Chen et al., 2015). Eight items from the original measure were not included in this study because they did not fit the context of the current study (e.g., “I feel excluded from the group I want to belong to”). As a result, the questionnaire contained six items of competence satisfaction (e.g., “I feel confident that I can do things well”), six items of autonomy satisfaction (“I feel I have been doing what really interests me”), and four items of relatedness satisfaction with the teacher (“I experience a warm feeling toward the teacher”). A higher score indicated more satisfaction of the particular need (αs = 0.78, 70, 67^2^ for competence, autonomy, and relatedness).

#### Language Mindset Inventory

We used the Language Mindset Inventory to assess participants’ fixed and growth mindsets about language learning ability (Lou and Noels, 2017). Participants rated their agreement on nine growth mindset items (“How good you are at using a foreign language will always improve if you really work at it”) and nine fixed mindset items (e.g., “You have a certain amount of language intelligence, and you can’t really do much to change it”) on a six-point scale (1 = Strongly Disagree to 6 = Strongly Agree). Given that fixed and growth mindsets are strongly and negatively correlated (*r* = −0.65, *p* < 0.001), growth mindset items were reversed coded such that a higher score represents a stronger fixed mindset (vs. growth mindset; α = 0.86).

#### Beliefs About Mistakes

We used the five-item Beliefs about Mistakes measure to tap participants’ beliefs about the importance of making mistakes in the English test (e.g., “I can develop new skills by making errors in the English test”; Tulis et al., 2018). The scale ranged from 1 (strongly disagree) to 6 (strongly agree). A higher score represents a more positive belief about making mistakes (α = 0.89).

#### Future Task Avoidance

We adapted Elliot and Church’s (1997) measure of performance-avoidance orientation to measure participants’ avoidance orientation toward partaking in another similar English test. This set of questions contained five items (e.g., I am thinking, “I will try to avoid doing this task again”; “I am worried that I may look incompetent if I do the test again”). Participants responded on a scale from 1 (not at all true of me) to 5 (very true of me). A higher score indicates a stronger avoidance tendency for a possible future task (α = 0.84).

#### Language Confidence

Participants rated their English confidence on a six-item measure (e.g., “I feel confident using English regardless of my ability”; Clément and Kruidenier, 1985). Participants responded on a six-point scale from 1 (totally disagree) to 6 (totally agree). A higher score indicates stronger language confidence (α = 0.94).

#### Duration of Task Engagement

The computer automatically recorded the time (in seconds) that participants spent on reviewing the answer keys. Given the time was not normally distributed (*M* = 79.44, *SD* = 90.81, skewness = 3.92, kurtosis = 23.57), we log-transformed this variable (Table 1).

## Results

### Preliminary Analysis

We found that participants performed poorly in the test; the average correct answer was 3.80 (*SD* = 2.02) out of 13 questions (29%). Moreover, participants in the three conditions did not differ significantly in their test scores, *F*(2,177) = 2.26, *p* = 0.11, suggesting that participants’ pre-experiment competence was equivalent across conditions. All self-report variables and the log-transformed variable of the task duration supported the normality assumption (Table 1). We found that participants in the three conditions showed no significant differences in terms of their year of living in Canada, *F*(2,77) = 1.33, *p* = 0.27, and gender distribution, χ^2^(2) = 1.24, *p* = 0.54. We also found that gender and year of living in Canada did not predict meta-lay theories. However, we found that men are more satisfied with their confidence (*M* = 3.47, *SD* = 0.74) compared to women (*M* = 3.17, *SD* = 0.79; *t* = –2.59, *df* = 178, *p* = 0.01) and that those who lived in Canada longer are more satisfied with their confidence (*r* = 0.17, *p* = 0.02) and autonomy (*r* = 0.21, *p* = 0.006) and felt more confidence (*r* = 0.32, *p* < 0.001). Because gender and length of residence did not change the conclusions of the major findings, we did not include these variables in further analysis (see Supplementary Data Sheet 1 for the results that include gender and length of residence).

### Correlation

As shown in Table 1, meta-lay theories are correlated with a sense of competence, autonomy, and relatedness, as well as with language mindsets and beliefs about mistakes. Those who felt that the teacher did not believe in their potential (i.e., a fixed meta-lay theory) were also less satisfied with their competence, autonomy, and relatedness and also more likely to endorse fixed (vs. growth) mindsets about L2 ability and negative beliefs about making mistakes. We also found that the correlations between mindsets and need satisfaction were significant and moderate (*r* = -0.25 to -0.30, *p*s < 0.001), as well were the correlations among the three aspects of need satisfaction (*r* = 0.34 to 0.55, *p*s < 0.001). Additional analyses indicated no multicollinearity issues between mindsets and the three aspects of need satisfaction on any outcome variable [variance inflation factors (VIFs) ≤ 1.50]. Moreover, we found that meta-lay theories were not correlated with any outcome variable, but mindsets and need satisfaction were significantly correlated with beliefs about mistakes, future task avoidance, and language confidence (| *r*| s ≥ 0.23, *p* < 0.01). That is, those who endorsed growth mindsets and those who felt more satisfied with the psychological needs were more likely to hold positive beliefs about making mistakes, less likely to avoid future tasks, and felt more confident about using English. However, satisfaction with competence and relatedness were the only two variables that positively correlated with the duration of task engagement (*r*s = 0.17 and.25, *p* < 0.05).

### Main Effect of the Feedback Manipulations

**FIGURE 2:**
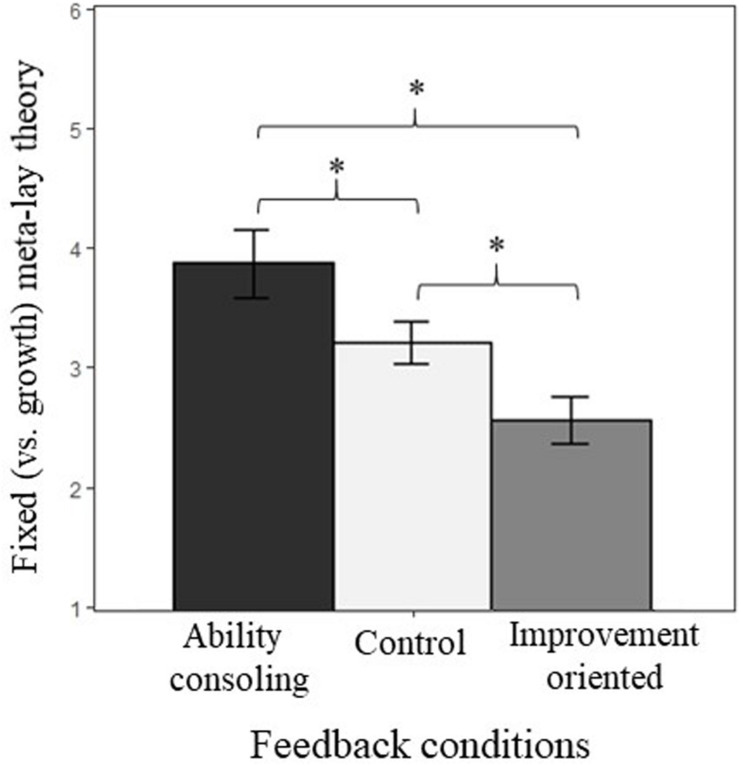
Mean meta-perceptions of potential across the three conditions. Error bars represent 95% confidence intervals.

One-way ANOVA supported Hypothesis 1 and suggested that the quality of ability feedback had a strong influence on meta-lay theories (Table 2), *F*(2,184) = 34.71, *p* < 0.001, η_*p*_^2^ = 0.28 (strong effect size). Tukey *post hoc* tests showed that participants in the consoling feedback condition perceived that the teacher believed the participants’ ability was less likely to be improved (*M* = 3.20, *SD* = 0.98) than participants in the improvement condition (*M* = 2.06, *SD* = 0.61), *p* < 0.001, and participants in the control condition were midway between the other two groups and differed significantly from both (*M* = 2.73, *SD* = 0.56; Figure 2), *p*s ≤ 0.002.

**TABLE 2:** The effect of feedback conditions (mean differences) on outcome variables.

Outcome variables	Condition	*M*	*SD*	*95% CI*		*F*	*p*	η_*p*_^2^
				Lower	Upper			
Meta-lay theories (fixed vs. growth)	Consoling	3.20^a^	0.98	2.95	3.45	34.71***	< 0.001	0.28
	Control	2.73^b^	0.56	2.59	2.87			
	Improvement	2.06^c^	0.61	1.90	2.22			
Competence	Consoling	3.04^b^	0.78	2.84	3.24	5.12**	0.007	0.06
	Control	3.38^a^	0.70	3.20	3.56			
	Improvement	3.46^a^	0.81	3.24	3.68			
Autonomy	Consoling	3.19	0.68	3.01	3.36	2.74	0.068	0.03
	Control	3.30	0.64	3.14	3.47			
	Improvement	3.48	0.70	3.29	3.66			
Relatedness	Consoling	3.73	0.67	3.56	3.90	2.30	0.104	0.03
	Control	3.78	0.61	3.62	3.93			
	Improvement	3.97	0.63	3.80	4.14			
Language mindsets	Consoling	2.84	0.66	2.67	3.01	1.10	0.334	0.01
	Control	2.77	0.63	2.61	2.93			
	Improvement	2.66	0.71	2.47	2.85			
Beliefs about mistakes	Consoling	4.43	0.95	4.19	4.67	0.90	0.409	0.01
	Control	4.54	0.94	4.30	4.78			
	Improvement	4.65	0.81	4.44	4.87			
Future task avoidance	Consoling	3.14^a^	1.03	2.88	3.40	3.34*	0.038	0.04
	Control	2.67^b^	0.94	2.43	2.91			
	Improvement	2.87^ab^	1.11	2.57	3.17			
Language confidence	Consoling	4.41	1.19	4.11	4.71	0.41	0.667	0.01
	Control	4.53	1.00	4.27	4.78			
	Improvement	4.58	0.89	4.34	4.81			
Duration of task engagement	Consoling	1.77	0.35	1.68	1.86	2.10	0.118	0.02
	Control	1.65	0.40	1.54	1.75			
	Improvement	1.78	0.39	1.68	1.89			

***p < 0.001, **p < 0.01, and *p < 0.05. ^abc^The same superscript represent no significant different between groups, Tukey’s honestly significant difference (HSD) test. ns = 62, 62, and 56 for consoling, control, and improvement feedback, respectively.

We also explored whether feedback conditions directly affected mindsets and need satisfaction. As shown in Table 2, feedback directly affected a sense of competence, but not relatedness, autonomy, or language mindsets. Specifically, participants in the consoling condition had a lower sense of competence than participants in the control and improvement conditions. However, there were no significant differences between control and improvement conditions.

Finally, we found that the feedback type influenced task avoidance, but did not influence other outcome variables. Specifically, participants in the consoling condition reported that they were more likely to avoid future tasks than did participants in the control condition. However, there was no significant difference between participants in the improvement condition and control condition.

### Path Analyses

**FIGURE 3:**
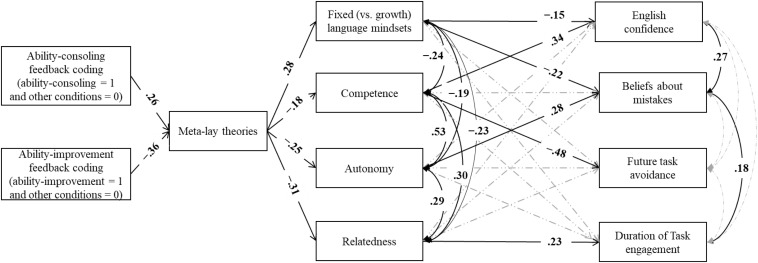
Results of the final path model. Numbers are standardized path coefficients. The solid dark lines represent significant paths (*p* < 0.05); the dashed gray lines represent non-significant paths.

To test the hypothesized model presented in Figure 1 and the five hypotheses holistically, we used Mplus 8.0 (Muthén and Muthén, 2010) to conduct a path analysis of the direct and mediated effects. Given that the independent variables were multi-categorical, feedback was dummy coded, following the recommendation of Hayes and Preacher (2014): Ability-consoling feedback (1 = consoling feedback vs. 0 = no feedback and improvement feedback) and improvement-oriented feedback (1 = improvement feedback vs. 0 = no feedback and consoling feedback). That is, the no-feedback condition was coded as the reference condition and was compared to the other two feedback conditions. The hypothesized model fit the data well [χ*^2^* = 27.85, *df* = 20, *p* = 0.11, comparative fit index (CFI) = 0.98, root mean square error of approximation (RMSEA) = 0.047, 90% CI = 0.00-0.08, and standardized root mean square residual (SRMR) = 0.035]. The results of the standardized path coefficients are presented in Figure 3, and unstandardized path coefficients are presented in Table 3. To understand the mediation effects, we used a bootstrapping resampling method to test the indirect effects (Hayes and Preacher, 2014). The results of the indirect effects are presented in Table 4.

**TABLE 3:** Unstandardized path coefficients of the path model.

Outcome variable	Predictor	*b*	*SE*	*t*	*p*	95% CI	*R*^2^
Meta-lay theories	Consoling feedback	0.47***	0.13	3.54	< 0.001	0.210, 0.731	0.28
	Improvement feedback	−0.67***	0.14	–4.92	< 0.001	−0.940, −0.405	
Language mindsets	Meta-lay theories	0.22***	0.05	3.95	< 0.001	0.108, 0.321	0.08
Competence		−0.16*	0.07	–2.42	0.015	−0.287, −0.030	0.03
Autonomy		−0.20***	0.06	–3.52	< 0.001	−0.307, −0.087	0.06
Relatedness		−0.23***	0.05	–4.43	< 0.001	−0.332, −0.128	0.10
Language confidence	Language mindsets	−0.22*	0.11	–2.09	0.037	−0.435, −0.014	0.25
	Competence	0.44***	0.10	4.24	< 0.001	0.239, 0.650	
	Autonomy	0.14	0.12	1.19	0.234	−0.092, 0.378	
	Relatedness	0.14	0.12	1.18	0.239	−0.090, 0.363	
Beliefs about mistake	Language mindsets	−0.30**	0.10	–3.11	0.002	−0.489, −0.111	0.21
	Competence	0.08	0.09	0.84	0.401	−0.105, 0.264	
	Autonomy	0.37***	0.11	3.44	0.001	0.159, 0.581	
	Relatedness	0.07	0.10	0.65	0.513	−0.136, 0.271	
Future task avoidance	Language mindsets	0.14	0.10	1.40	0.163	−0.058, 0.345	0.32
	Competence	−0.64***	0.10	–6.35	< 0.001	−0.833, −0.440	
	Autonomy	–0.11	0.11	–0.97	0.332	−0.336, 0.114	
	Relatedness	–0.04	0.11	0.38	0.705	−0.259, 0.175	
Duration of task engagement	Language mindsets	0.03	0.04	0.78	0.434	−0.052, 0.120	0.07
	Competence	0.06	0.04	1.28	0.200	−0.029, 0.139	
	Autonomy	–0.01	0.05	–0.14	0.891	−0.103, 0.089	
	Relatedness	0.14**	0.05	2.90	0.004	0.044, 0.230	

*p < 0.05, **p < 0.01, and ***p < 0.001.

**TABLE 4:** Indirect effects for the path model: estimates, standard error (SE), and 95% bias-corrected confidence intervals (CIs).

Hypothesis	Parameter	Estimate	*SE*	Lower 2.5% CI	Upper 2.5% CI	Effect size
H4	Consoling feedback Meta-lay theories Language mindsets	0.10*	0.04	0.040	0.192	0.07
	Consoling feedback→Meta-lay theories→Competence	−0.08*	0.04	–0.172	–0.011	0.05
	Consoling feedback→Meta-lay theories→Autonomy	−0.09*	0.04	–0.192	–0.026	0.07
	Consoling feedback→Meta-lay theories→Relatedness	−0.11*	0.04	–0.202	–0.025	0.08
	Improvement feedback→Meta-lay theories→Language mindsets	−0.14*	0.06	–0.269	–0.056	0.10
	Improvement feedback→Meta-lay theories→Competence	0.11*	0.05	0.018	0.221	0.06
	Improvement feedback→Meta-lay theories→Autonomy	0.16*	0.05	0.077	0.257	0.11
	Improvement feedback→Meta-lay theories→Relatedness	0.13*	0.05	0.056	0.232	0.09
H5(a)	Consoling feedback→Meta-lay theories→Mindsets→Confidence	−0.02*	0.01	–0.078	–0.001	0.01
	Consoling feedback→Competence→Mindsets→Confidence	−0.03*	0.02	–0.098	–0.005	0.02
	Improvement feedback→Meta-lay theories→Mindsets→Confidence	0.03*	0.02	0.001	0.090	0.02
	Improvement feedback→Competence→Mindsets→Confidence	0.05*	0.03	0.010	0.115	0.02
H5(b)	Consoling feedback→Meta-lay theories→Mindsets→Beliefs about mistakes	−0.03*	0.02	–0.076	–0.009	0.02
	Consoling feedback→Meta-lay theories→Autonomy→Beliefs about mistakes	−0.03*	0.02	–0.099	–0.007	0.02
	Improvement feedback→Meta-lay theories→Mindsets→Beliefs about mistakes	0.04*	0.02	0.011	0.114	0.02
	Improvement feedback→Meta-lay theories→Autonomy→Beliefs about mistakes	0.05*	0.03	0.013	0.126	0.03
H5(c)	Consoling feedback→Meta-lay theories→Competence→Future task avoidance	0.05*	0.03	0.007	0.120	0.02
	Improvement feedback→Meta-lay theories→Competence→Future task avoidance	−0.07*	0.03	–0.148	–0.012	0.03
H5(d)	Consoling feedback→Meta-lay theories→Relatedness→Duration of engagement	−0.02*	0.01	–0.036	–0.004	0.02
	Improvement feedback→Meta-lay theories→Relatedness→Duration of engagement	0.02*	0.01	0.007	0.045	0.03

*A 95% biased-corrected CI (with 5,000 bootstrap samples) not including zero indicates significant indirect effects. The effect sizes are the absolute values of the standardized estimates of the respective path coefficient.

#### Feedback Influences Meta-Lay Theories (Hypothesis 1)

We found that both contrasts of feedback significantly predicted meta-lay theories. Specifically, consoling feedback (vs. improvement feedback and no feedback) positively predicted meta-lay theories, whereas improvement feedback (vs. consoling feedback and no feedback) negatively predicted meta-lay theories. This finding is consistent with the ANOVA findings in Table 2.

#### Meta-Lay Theories Predict Mindsets and Need Satisfaction (H2)

Supporting Hypothesis 2, meta-lay theories predicted language mindsets and satisfaction of the three psychological needs. Those who strongly perceived their teacher believes their ability to be fixed also more likely to endorse fixed mindsets about their own language ability (β = 0.28, *p* < 0.001), have a lower sense of competence (β = -0.18, *p* = 0.014), relatedness (β = -0.31, *p* < 0.001), and autonomy (β = -0.25, *p* < 0.001). These findings support the claim that language mindsets and psychological need satisfaction are both predicted by the meta-perceptions.

#### Mindsets and Need Satisfactions Jointly Predict Motivational Outcomes (Hypothesis 3)

In the path analysis, we also found that fixed (vs. growth) language mindsets were negatively and weakly associated with perceived competence (β = -0.24, *p* = 0.001), autonomy (β = -0.19, *p* = 0.010), and relatedness (β = -0.23, *p* = 0.001). Those who felt more satisfied with their psychological needs also more likely to endorse a stronger growth mindset. These findings support the hypothesis that mindsets and satisfaction of psychological needs were interrelated but distinct constructs.

Regarding their joint predictions on motivational outcomes, we found that language mindsets (β = -0.15, *p* = 0.035) and perceived competence (β = 0.34, *p* < 0.001) jointly predicted confidence to use English. Language mindsets (β = -0.22, *p* = 0.002) and perceived autonomy (β = 0.28, *p* < 0.001) jointly predicted beliefs about making mistakes. These findings showed that confidence and the beliefs about making mistakes were predicted by perceived competence and autonomy, respectively, as well as by mindsets. This finding highlights the independent but complementary contribution of some aspects of need satisfaction and mindsets to the prediction of motivational variables. However, we found that only perceived competence predicted future task avoidance (β = -0.48, *p* < 0.001), and only perceived relatedness predicted the duration of engagement with the task (β = 0.22, *p* = 0.005). Although language mindsets were significantly correlated to future task avoidance (*r* = 0.25, *p* < 0.001), this link was no longer significant in the path model (β = 0.09, *p* = 0.162).

#### The Effect of Feedback on Mindsets and Need Satisfaction Through Meta-Lay Theories (Hypothesis 4)

We tested whether feedback indirectly influenced language mindsets and need satisfaction through fixed (vs. growth) meta-lay theories. As shown in Table 4, consoling feedback resulted in a higher score in meta-lay theories, which in turn predicted stronger fixed mindsets, and a lower sense of competence, autonomy, and relatedness relative to no feedback and improvement feedback. In contrast, improvement feedback led to a lower score in meta-lay theories, which in turn predicted stronger endorsement of growth language mindsets, and a higher sense of competence, autonomy, and relatedness relative to no feedback and consoling feedback. In summary, these findings supported that feedback indirectly influenced language mindsets and need satisfaction through meta-lay theories.

#### Feedback Influenced Outcomes Through Meta-Lay Theories, Mindsets, and Psychological Need Satisfaction (Hypothesis 5)

We tested whether feedback indirectly predicted motivational outcomes (Table 4, H5a to H5d). First, we found that consoling feedback negatively and improvement feedback positively influenced English confidence through meta-lay theories and then mindsets and a sense of competence (see H5a). Second, consoling feedback negatively and improvement feedback positively influenced beliefs about mistakes through meta-lay theories and then mindsets and a sense of autonomy (see H5b). Third, consoling feedback positively and improvement feedback negatively predicted future task avoidance through meta-perceptions and then competence (H5c). Finally, consoling feedback negatively and improvement feedback positively predicted the duration of task engagement through meta-lay theories and then relatedness (H5d). In summary, the feedback type influenced different outcomes through meta-lay theories and then mindsets and/or need satisfaction.

## Discussion

English-as-a-second-language learners are sensitive to subtle interpersonal signals that indicate whether other people in their social and learning environments believe they are capable or not, which can impact their motivation to use English. In this study, ESL learners experienced challenges in an English test, and one group of learners received ability-consoling feedback, the second group received improvement feedback, while the third group received no additional feedback (i.e., control group). We found that compared to learners who received no feedback, those who received improvement-oriented feedback perceived that their teacher believed that they could improve their ability (i.e., growth meta-lay theory). In contrast, compared to learners who received no feedback, those who received ability-consoling feedback perceived the teacher believed less in their potential to improve and had a weaker sense of competence in English. Furthermore, we identified two pathways through which feedback and meta-lay theories predicted motivational outcomes: the path through mindsets and the paths through need satisfaction. That is, meta-lay theories predict learners’ growth (vs. fixed) mindsets and their need satisfaction, which can in turn influence important motivational outcomes, including learners’ willingness to retake the English test they failed, confidence in using English, and the time they spend on reviewing the answer keys. As such, both meaning-making processes about growth (i.e., mindsets) and sense of need satisfaction are important for learners’ resilience in challenging situations (Lou and Noels, 2019a).

### Theoretical Contributions

The findings contribute to bridging two important motivation theories, SDT and MT, in three ways. First, we found that learners’ mindsets were only weakly linked to their sense of competence, autonomy, and relatedness, suggesting that mindsets and need satisfactions are related but distinct concepts. Learners who endorsed growth mindsets were more likely to feel they are capable, have choices in their learning, and related to the feedback provider. Second, we found that the quality of feedback is an important social factor that influenced both mindsets and need satisfaction either directly or indirectly through meta-lay theories. These findings extended previous research on how others’ feedback influences learners’ mindsets (Rattan et al., 2012) and are consistent with the notion of perceived need support from SDT (Ryan and Deci, 2020). Specifically, feedback directly influenced a sense of competence, but indirectly influenced growth mindsets, and feelings of relatedness and autonomy through meta-lay theories. Third, we found that mindsets and need satisfaction jointly predicted adaptive outcomes. When controlling for the correlations between feelings of satisfaction of the three needs and mindsets, learners’ own mindsets accounted for distinct variance in English use confidence and beliefs about mistakes. Similarly, a sense of competence contributed uniquely to English use confidence and future task avoidance, a sense of autonomy contributed uniquely to beliefs about mistakes, and a sense of relatedness contributed uniquely to the length of time learners spent reviewing their mistakes. Together, these findings suggest that combining MT (Dweck et al., 1995) with SDT (Deci and Ryan, 2000) can enrich the understanding of how interpersonal factors predict learners’ responses to failure situations—through both meaning-making about ability and the sense of psychological need satisfaction.

Our study also contributes to the growing research on how learners’ growth mindsets are developed (e.g., Haimovitz and Dweck, 2017; Rattan et al., 2018). Previous research showed that teachers’ and parents’ self-reported mindsets do not predict learners’ mindsets; rather, the way that teachers and parents react to learners’ failures predicted learners’ mindsets (Park et al., 2016; Haimovitz and Dweck, 2017). Our study further suggests that teachers’ instructional practices indirectly impact mindsets through learners’ perceptions of the teachers’ beliefs. As such, one way that learning climates and teaching practices can help learners to adopt a growth mindset is through learners’ perceptions that their teachers believe in their potential. Thus, we argue that if teachers’ and parents’ mindsets are transmitted to learners explicitly or implicitly through the type of feedback they offer, students will develop and adjust their own mindsets in line with how they perceive significant other people to view them. This process, whereby perceptions of others’ beliefs about the learners’ potential function as a mirror through which learners see their own ability, may have far-reaching effects on learners’ achievement and persistence. For example, learners’ who developed growth mindsets are more resilient in failure situations (Yeager and Dweck, 2012). In language learning, research also found that those with growth mindsets are less likely to give up language learning and feel less anxious when using the target language (Lou and Noels, 2016, 2019c). They are also more likely to use the language outside the classroom (Lou and Noels, 2020).

### Limitations and Future Directions

Like other lab-based experiments, the results of this experiment may not be generalizable to actual classrooms given that the “teacher” in this study was not actually a trained teacher, and the communication between the participants and the “teacher” may not reflect classroom dynamics. However, because ESL learners often receive feedback about their language competence from many different interlocutors (not just teachers), their responses in this lab interaction may reflect their natural reactions to receiving different feedback from native speakers. Moreover, this randomized controlled experiment serves an important step for clarifying concepts and mechanisms to inform the development of “real-world” research. Building on this current research, future longitudinal field experiments might address how receiving different kinds of feedback changes meta-lay theories for learners in the language classroom and the long-term effects of providing learners with growth meta-lay theories.

In this study, we only focused on students’ reactions to two possible types of feedback to their failure (i.e., ability-consoling and improvement-oriented). Future research could investigate whether and how other aspects of interpersonal feedback influence learners’ need satisfaction and mindsets. For example, research suggests that autonomy support versus being controlling and well-structured (e.g., clear expectations and explicit directions in learning) versus a chaotic environment can affect need satisfaction (Reeve and Jang, 2006; Jang et al., 2016). However, the impact of different aspects of autonomy-supportive strategies on mindsets has not been examined. Similarly, research suggested that a performance-oriented environment (e.g., competition for grades) versus a learning-oriented environment (Leith et al., 2014), generic statements (e.g., “boys are always good at math”) versus specific statements (e.g., “That student is good at math”; Cimpian, 2010), and ability praise (“You are so smart”) versus process praise (e.g., “Good Job, you worked so hard”; Pomerantz and Kempner, 2013) can strengthen fixed mindsets (see Haimovitz and Dweck, 2017; Muenks et al., 2020). Although these antecedents of mindsets may share overlap with autonomy-support strategies, they have not been systematically studied in reference to the SDT literature. In addition, a student may believe that their teacher believes their ability is fixed or malleable through various non-verbal cues, such as the teachers’ tone of enthusiasm (Young-Jones et al., 2014). Future research would also benefit from observing teachers’ behaviors to identify what other strategies teachers use in the classroom to shift learners’ meta-lay theories and enhance learners’ growth mindsets and/or need satisfaction. Finally, in addition to the duration of reviewing answer keys, future research may include students’ revision and follow-up performance to understand the role of mindsets in learning behaviors and outcomes (cf. Cutumisu and Lou, 2020).

To further extend the integration of MT and SDT, future research could also study different domains, learning situations, and outcomes that are more commonly studied in SDT but not MT research, and vice versa. In this study, we focused on the domain of English language learning, particularly in the face of a challenging situation. Given that SDT and MT are general motivational theories that have been applied to different educational domains (Dweck and Yeager, 2019; Ryan and Deci, 2020), integrating mindsets and SDT may benefit motivation research in other areas. In addition, meta-lay theories are relevant not only in challenging situations. For example, in competitive situations where ability is emphasized and successful situations where teachers praise learners’ intelligence, learners may draw on their meta-lay theories and thereby influence their motivation. In these different situations, it would be important to examine whether the integrated model can better explain learners’ emotional, behavioral, and achievement development than either SDT or MT alone. For example, previous meta-analytical research found a consistent but small effect of mindsets on achievement (Sisk et al., 2018). Similarly, mindsets was found to have little influence on language performance (Chaffee et al., 2020). Applying the integrated model, future research can continue to identify unique and overlapping effects of different growth-related constructs on learning engagement and achievement, which can have implications for designing more effective and parsimonious interventions.

### Pedagogical Implications

Providing feedback about learners’ ability is one of the most powerful tools to help learners cultivate confidence and regulate their behavior to achieve their goals (Koenka et al., 2019). However, it is inevitable that learners will receive negative feedback when they fail. This study supports the idea that failures are less detrimental when teachers provide growth-oriented feedback and are more detrimental when teachers focus on learners’ innate ability (cf. Rattan et al., 2012; Skipper and Douglas, 2015; Fong et al., 2019). We further learned that how teachers communicate their feedback about the learners’ abilities can shape learners’ motivation through their perceptions about the teachers’ beliefs. That is, teachers’ feedback may be most effective in encouraging “growth” when teachers make learners feel that the teachers believe in the learners’ potential to improve. To do so, educators should first pay attention to their own beliefs (Heyder et al., 2019) and how they communicate their beliefs to learners. Previous research showed that teachers who believed in fixed mindsets are more likely to create a more controlling learning environment (Leroy et al., 2007; Canning et al., 2019) and to provide consoling feedback to poor performers (Rattan et al., 2012). Although consoling feedback may seem intuitively positive and consistent with the theory of multiple intelligences, our findings showed that comforting learners who failed in a given domain (e.g., languages) by assuring them that they are good in other domains could lead to negative motivational consequences. Thus, teachers should be mindful of how such beliefs may impact their practices and learners’ motivation.

Learners’ mindsets and need satisfaction are easily influenced by significant others in different ways (e.g., praise, guided attribution, competition; Leith et al., 2014; Haimovitz and Dweck, 2017). As this study showed, learners could readily perceive whether the feedback provider believed in their potential in English or not, which in turn influenced learners’ psychological need satisfaction and mindsets. To foster a growth mindset in their learners, for example, educators can openly share their belief that everyone has the potential (Rattan et al., 2018), provide opportunities for learners to experience “growth,” and behave in line with their beliefs (e.g., by providing improvement feedback, creating a fair learning environment that does not favor high-achieving learners, utilizing growth-oriented assessments, and providing support when it is needed; see Lou and Noels, 2019b, for a discussion). As a result, learners who perceive that their teachers support their growth would likely put more effort, feel more confident, and be more resilient in the face of challenges (Burns et al., 2019; Dweck and Yeager, 2019).

## Conclusion

This study demonstrated that meta-lay theories are an important interpersonal perception that underlies the process by which others’ feedback influences learners’ motivation. As such, providing ability-consoling feedback can make learners think that the feedback provider does not believe in the learners’ potential and that they are not competent, which can lead to negative motivational consequences, including spending less time reviewing test materials, unwillingness to redo the test, and a lack of language confidence. In contrast, providing improvement-oriented feedback can lead to positive effects by shifting learners’ perceptions that the feedback provider believes in the learners’ potential. Moreover, we extend previous research through the findings that mindsets and need satisfaction have independent yet complementary effects on motivational outcomes, hence providing support for integrating MT and SDT.

## Data Availability Statement

The datasets generated for this study are available on request to the corresponding author.

## Ethics Statement

The studies involving human participants were reviewed and approved by University of Alberta Ethics Board. The patients/participants provided their written informed consent to participate in this study.

## Author Contributions

NL and KN conceived the idea, developed the materials, and contributed to the interpretation of the results. NL carried out the experiment and took the lead in writing the manuscript. KN provided critical feedback and helped shape the research, analysis, and manuscript. All authors contributed to the article and approved the submitted version.

## Conflict of Interest

The authors declare that the research was conducted in the absence of any commercial or financial relationships that could be construed as a potential conflict of interest.

## Footnotes

1 The sample size of 180 is more than the suggested minimum sample size of 100 for path analysis based on the ratio of 10 cases per variable (10 variables in this study; Kline, 2015). Moreover, a power analysis based on a previous experimental study concerning the effects of feedback on students’ perceptions of teacher (Rattan et al., 2012; Study 3; η^2^ = 0.38) suggested that detecting a significant effect size requires a sample of 39 participants (α = 0.05, power = 0.99, groups = 3). Thus, this current sample size is sufficient to detect the effect of feedback on meta-lay theories.

2 Deleting any items would not improve the α for relatedness. The average inter-item correlation of this measure (*r* = 0.33), which is another way of examining internal consistency, showed that the measure was within the ideal range (i.e., 0.20–0.40; Cohen and Swerdlik, 2005).
