# Supplementary material for: “Does My Teacher Believe I Can Improve?”: The Role of Meta-Lay Theories in ESL Learners’ Mindsets and Need Satisfaction
Source: Front Psychol. 2020 Aug 7;11:1417. doi: 10.3389/fpsyg.2020.01417 (PMC7426522; doi:10.3389/fpsyg.2020.01417)

Online Supplemental for:

"Does my teacher believe I can improve?": The role of meta-lay theories in ESL learners' mindsets and need satisfaction

Citation:

Lou, N. M, & Noels, K. A. "Does my teacher believe I can improve?": The role of meta-lay theories in ESL learners' mindsets and need satisfaction. *Frontiers in Psychology*. 11:1417. doi: 10.3389/fpsyg.2020.01417

Sections

1. Appendix A – Experimenter's Script
2. Appendix B – External Manipulation Check
3. Appendix C – GRE and LSAT Questions
4. Appendix D – Questionnaire
5. Appendix E – Participants' country of birth
6. Appendix F – Path model with gender and length of residence

Appendix A – Experimenter's Script

Hi there, thanks for coming in to participate with this study. This study is all about English ability and learning. My name is (experimenter's name), by the way. I'm an English teacher and I'm currently working on a master's degree in Education, and I was asked by the Department of Psychology to help out with the study since it is about an English test.

The study itself consists of two parts: an English test and a questionnaire. The English test is exactly 15 minutes long. It's all multiple choice. After the English test, you will receive your score on the computer. I will receive your score on my computer as well. After you get the feedback is when you can start the questionnaire.

As for the questionnaire, keep in mind that I don't have access to your responses. I only have access to the scores for the English test. So please try to answer the questions as best as you can.

Make sure to read all instructions carefully. Also, if there is anything in the questionnaire that is unclear in terms of the working or phrasing, please let me know. Do you have any questions?

Appendix B – External Manipulation Check Questions

Condition 1: Imagine you were a student learning English as a second language. You failed an English test and your teacher provided you with the following feedback

> "I'm sorry that you did not do well on the test. I wanted to let you know that you're an adept and capable student. English isn't a subject for everyone—it's okay if you didn't do as great as you hoped. Some people aren't naturally good at languages. But I'm sure you have great talent in other subjects. I care about how you're doing and feeling with this task, so if you have any questions, feel free to talk to me about the task or about language learning in general after the study."

Do you agree that the teacher's intention is to console the student for failing the test?
Totally disagree Neutral Totally agree
1 2 3 4 5

Do you agree that the teacher's intention is to encourage the student to improve?
Totally disagree Neutral Totally agree
1 2 3 4 5

Condition 2: Imagine you were a student learning English as a second language. You failed an English test and your teacher provided you with the following feedback

> "I'm sorry that you did not do well on the test. I wanted to let you know that you're an adept and capable student. Like with many things, practice makes perfect. If you put in the work, you'll be at the level and proficiency that you want, so keep working on it. I care about how you're doing and feeling with this task, so if you have any questions, feel free to talk to me about the task or about language learning in general after the study."

Do you agree that the teacher's intention is to console the student for failing the test?
Totally disagree Neutral Totally agree
1 2 3 4 5

Do you agree that the teacher's intention is to encourage the student to improve?
Totally disagree Neutral Totally agree
1 2 3 4 5

Appendix C – GRE and LSAT Questions

Due to copy right, we did not include GRE and LSAT questions in this online supplement.

Please contact the author if needed.

Appendix D – Questionnaire

Meta-perceptions of potential (adapted from Rattan et al., 2019; Dweck, 1999)

Indicate the extent in to which you think about the English teacher by rating the agreement to the following statement from 1 = strongly disagree to strongly agree.

1	2	3	4	5	6
Strongly Disagree	Moderately Disagree	Slightly Disagree	Slightly Agree	Moderately Agree	Strongly Agree

*1. The teacher believes that I have a certain amount of language intelligence, and I can't really do much to change it.
*2. The teacher believes that language intelligence is something about me that I can't change very much.
*3. The teacher believes that I can't really change my language intelligence
4. The teacher believes that I can significantly change my language intelligence level
5. The teacher believes that I can always improve my foreign language ability.
6. The teacher believes that I can do well in a foreign language if I try hard.
Note. * These items are reversed coded. All items are randomized.

Basic Psychological Need Satisfaction (Chen et al., 2015).

Below, we are going to ask about your experiences of certain feelings. Indicate with a number between 1 (strongly disagree) and 5 (strongly agree) your agreement with each of the statements below.

Competence

I feel confident that I can do things well.
I feel capable at what I do.
I feel I can successfully complete difficult tasks.
*I have serious doubts about whether I can do things well.
*I feel insecure about my abilities.
*I feel like a failure because of the mistakes I make.

Autonomy

I feel a sense of choice and freedom in the things I undertake.
I feel that my decisions reflect what I really want.
I feel I have been doing what really interests me.
*Most of the things I do feel like "I have to".
*I feel forced to do many things I wouldn't choose to do
*I feel pressured to do too many things.

Relatedness

I feel that the teacher cared about me.
I experience a warm feeling towards the teacher.
*I feel that the teacher was cold and distant towards me.
*I have the impression that the teacher dislikes me.

Language Mindsets Inventory (Lou & Noels, 2017)

Instructions: Below are a number of statements about language intelligence, language intelligence is the capacity to use spoken and written language, including your native language and perhaps other languages, to express what's on your mind and to understand other people. People with high language intelligence display a facility with words and languages. They are typically good at reading, writing, telling stories.

Please rate how much you personally agree or disagree with these statements. There is no right or wrong answer. We are interested in your ideas.

1	2	3	4	5	6
Strongly Disagree	Moderately Disagree	Slightly Disagree	Slightly Agree	Moderately Agree	Strongly Agree

1. To be honest, you can’t really change your language intelligence.
2. Your language intelligence is something about you that you can’t change very much.
3. You have a certain amount of language intelligence, and you can’t really do much to change it.
4. *You can always improve your language intelligence substantially.
5. *No matter who you are, you can significantly improve your language intelligence level.
6. *No matter how much language intelligence you have, you can always improve it quite a bit.
7. You can’t change how capable you are at learning new languages.
8. To be honest, you can’t really change your basic ability to learn and use new languages.
9. To a large extent, your ability to learn new languages is innate and you can’t change much.
10. *You can always improve how good you are at learning new languages.
11. *No matter who you are, you can always improve your basic ability to learn new languages.
12. *No matter how much ability you have in learning new languages, you can improve it considerably.
13. After a certain young age, you have very limited ability to learn new languages.
14. You don’t really have the ability to learn new languages after a certain young age.
15. Your ability to learn new languages is restricted after a certain young age, and you can’t really change it.
16. *No matter how old you are, you can always improve your ability to learn new languages.
17. *Regardless of age, you can significantly improve how good you are at learning new languages.
18. *Even after a certain young age, you can substantially improve your ability to learn new languages.

Note. * These items are incremental items. All items are randomized in the survey.

Beliefs about mistakes (Tulis et al., 2018)
The following section contains statements concerning people's thoughts about English learning. Please rate how you feel about the statements RIGHT NOW and rate your opinion on the scale below from 1 (strongly disagree) to 6 (strongly agree)

1. I can learn something from my mistakes/errors in the English test.
2. Mistakes/errors are important for getting better at the English test.
3. I can develop new skills by making errors in the English test.
4. My errors in the test help me to improve my skills in English.
5. Errors in the English test help me to be better at it later on.

Future tasks avoidance
Instructions: Imagine you were offered the opportunity to partake in the test again after this questionnaire. Indicate the extent to which each statement apply to you, from 1 (not at all true of me) to 5 (very true of me)

1. I will try to avoid doing this task again.
2. I am worried that I may look incompetent if I do the test again.
3. I am worried that the teacher may judge my English ability if I do the test again.
4. I am worried that if I fail the test again, the teacher might think I am not competent.
5. I am thinking, "What if I perform poorly again?"

Confidence (Clément and Kruidenier 1985**)**
Think about your English studies right now. Indicate the extent to which you agree with each item from 1 (strongly disagree) to 6 (strongly agree)

1. I may not be completely fluent in English, but I feel confident using it.
2. Despite the fact that I may not be completely proficient in English, I am confident using it.
3. Even when I make mistakes, I still feel sure of myself while trying to use English.
4. I am confident when using English despite any errors I may make.
5. Regardless of how much English I know, I feel confident about using it.
6. I feel confident using English regardless of my ability.

Appendix E – Participants' country of birth

Frequency	*Per cent*	Country of birth	*Frequency*	*Per cent*	Country of birth
70	38.9	China	1	.5	France
16	8.9	South Korea	1	.5	Italy
16	8.9	India	1	.5	Macedonia
6	3.3	Pakistan	1	.5	Romania
6	3.3	Philippines	1	.5	Turkey
5	2.8	Taiwan	1	.5	Egypt
4	2.2	Vietnam	1	.5	Syria
4	2.2	Russia	1	.5	Eritrea
4	2.2	Saudi Arabia	1	.5	Ethiopia
3	1.7	Bangladesh	1	.5	Ghana
3	1.7	Sri Lanka	1	.5	Kenya
3	1.7	Nigeria	1	.5	Peru
3	1.7	Colombia	4	2.2	Missing data
3	1.7	Cuba			
2	1.1	Nepal			
2	1.1	Serbia			
2	1.1	Sweden			
2	1.1	Ukraine			
2	1.1	Iraq			
2	1.1	Lebanon			
2	1.1	Congo			
1	.5	Indonesia			
1	.5	Singapore			
1	.5	Thailand			
1	.5	Belarus			

Appendix F

We added the paths of gender and length of residence on different outcomes based on the correlational results. The model fit the data well: $\chi^2 = 45.92$, $df = 34$, $p = .08$; CFI = .97; RMSEA = .04; SRMR = .04.

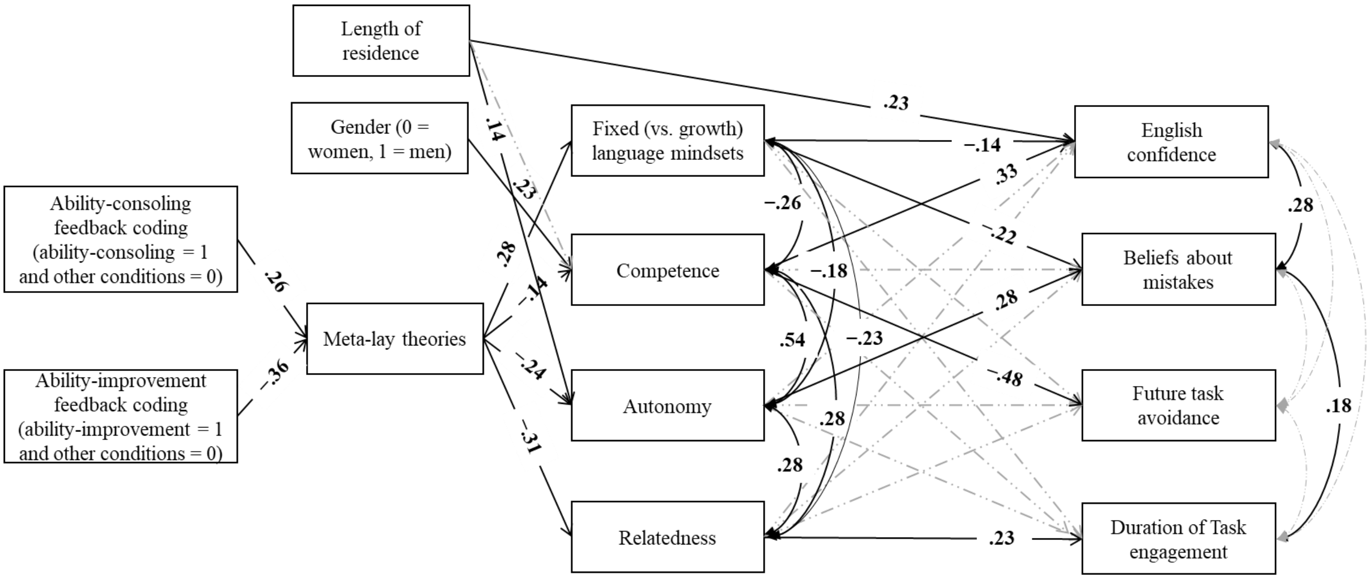

Supplement: Supplementary file 1 [file Data_Sheet_1.pdf]
